# Predictors of surgical site infections following cesarean delivery in public hospitals of West Ethiopia: A cohort study

**DOI:** 10.1371/journal.pone.0339930

**Published:** 2026-01-21

**Authors:** Mesfin Abera Hambisa, Meseret Belete Fite, Robsan Gudeta Getachew, Temesgen Tilahun, Dereje Chala, Emiru Merdasa, Befirdu Mulatu, Werku Etafa, Abdisa Abera, Misganu Teshoma Regasa

**Affiliations:** 1 Wollega University comprehensive specialized hospital, Institute of Health Sciences, Wollega University, Nekemte, Ethiopia,; 2 Department of Public Health, Institute of Health Sciences, Wollega University, Nekemte, Ethiopia; 3 Department of Obstetrics and Gynecology, School of Medicine, Institute of Health Sciences, Wollega University, Nekemte, Ethiopia; 4 Department of Nursing, Institute of Health Sciences, Wollega University, Nekemte, Ethiopia; 5 Department of Midwifery, Institute of Health Sciences, Wollega University, Nekemte, Ethiopia; Hospital Femina, BRAZIL

## Abstract

**Background:**

Surgical site infection rates after cesarean delivery among women who delivered are increasing at an alarming rate. This results in maternal mortality, disability, prolonged hospital stay, significant financial expense, psycho-social disturbance, antibiotic resistance, and a window period opportunity missed for the baby. Despite previous Ethiopian studies on surgical site infection after cesarean delivery with a cohort design being conducted in a single center and did not consider certain risk factors, which limited their findings. This study accounts for factors such as the American Society of Anesthesiologists (ASA) class, postoperative hemoglobin levels, presence of meconium, postpartum hemorrhage, and antibiotic prophylaxis. This study aimed to determine the incidence and predictors of surgical site infection post- cesarean delivery among women who gave birth in public hospitals in the East Wollega Zone, West Ethiopia.

**Methods:**

A hospital-based prospective cohort study was conducted in East Wollega public hospitals from 15^th^ January to 14^th^ April, 2024, involving 408 participants selected consecutively. We entered data using Epi-Data version 4.6 and exported to STATA version 14 for analysis. The Kaplan-Meier method was used to estimate surgical site infection. The Cox proportional hazard assumptions were checked using Schoenfeld residual statistics, while the Cox-Snell residual test was used to check the final model’s adequacy. Then, in a multi-variable analysis, variables with a p-value < 0.05 were declared as predictors of surgical site infection.

**Results:**

The incidence rate of SSI is 9.4/1000 person/day (95% CI: 7.5, 11.6). Presence of meconium (AHR = 2.37, 95% CI: 1.3, 4.12), emergency Cesarean delivery (AHR = 2.5, 95% CI: 1.16, 5.41), American Society of Anesthesiologists class ≥ III (AHR = 5.3, 95% CI: 2.8, 9.78), postoperative hemoglobin <11 g/dl (AHR = 1.78, 95% CI: 1.04, 3.06), and absence of antibiotic prophylaxis (AHR = 2.46, 95% CI: 1.4, 4.21) were identified as statistically significant predictors of surgical site infection among post cesarean delivery mothers.

**Conclusion:**

This study demonstrated a significant incidence density of surgical site infection within the study area. For the identified predictors, the authors recommend the need for target interventions particularly, the consistent administration of antibiotic prophylaxis and vigilant monitoring of patients with existing health complications or those undergoing emergency procedures to reduce the burden of surgical site infection in this area.

## Introduction

A surgical site infection (SSI) refers to an infection that develops after surgery at the site of the incision. These infections can range from superficial ones, affecting only the skin, to more severe cases involving deeper tissues, organs, or both [1]. It develops within thirty days of a surgical procedure including cesarean delivery at the incision site and/or deeper underling tissue spaces [2]. Despite advances in operating room protocols, instrument sterilization technologies, improved surgical technique, and the most effective infection prevention strategies, Surgical site infections continue to be a major cause of hospital-acquired infection [3]. Surgical site infection rates among women delivered with cesarean delivery ranged from 2.9% in Europe to 48.2% in Africa [2,4,5].

A study conducted in Tanzania found that the incidence density of surgical site infections (SSI) was 37.5 cases per 10,000 people per day [6]. Also, study done in Serbia’s found 4.5 cases per 1000 patient days incidence density [7]. According study conducted in Tunisia showed the incidence density of surgical site infection was 12.9 cases per 1000 days of hospitalization [8]. A study conducted in Ethiopia in specialized hospitals in the Amhara region found that the surgical site infection incidence density was 17.59 cases per 1000 person-days of observation [9]. Prospective cohort at the Debre Markos Referral Hospital study shows the incidence density of surgical site infection was 11.7 cases per 1000 person/ day [10].

Surgical site infection (SSI) is the most common complication following cesarean delivery, accounting for 3% of all maternal deaths. It significantly contributes to both maternal and infant morbidity and mortality, facilitates the spread of antibiotic resistance, and prolongs hospital stays, thereby increasing healthcare costs [11,12].

Surgical site infections also impact several aspects of the mother’s recovery, including increased pain, reduced mobility, delayed wound healing, prolonged antibiotic use, the need for additional surgery, and compromised health, all of which affect her ability to care for her infant [13]. Several studies have identified factors that increase the risk of surgical site infections following cesarean delivery, including high BMI, an ASA classification of 3 or higher, prolonged surgery duration, delayed or inadequate antibiotic prophylaxis (over one hour), emergency procedures, chorioamnionitis, advanced maternal age, maternal comorbidity, use of staples for skin closure, significant blood loss, limited antenatal care, smoking, prematurely ruptured membranes, hypertensive disorders, multiple gestation, prolonged labor, delayed operation following membrane rupture, frequent vaginal examinations, surgery performed by teaching services or non-consultant doctors in training, use of subcutaneous drains, anemia, and extended hospital stays [6,14–20].

Over the last decade, there has been a greater emphasis on establishing and improvement of hospital- and community-based infection prevention and control programs to decrease SSI [21]. In national and international settings, effective infection prevention activities have been established. Such strategies as improved operating room ventilation, sterilizing methods, barriers, surgical techniques, and the provision of antimicrobial prophylaxis, decontamination, preoperative washing, and nutrition enhancement are among them. However, surgical site infection continues to be a significant cause of morbidity, prolonging hospitalization and increasing mortality [22,23].

In some countries, hospitals are implementing various programs to track surgical site infections (SSI) after discharge, reduce infection rates, and prevent related complications. Post-discharge SSI surveillance and control are key strategies being employed. Recent studies conducted in various countries indicate that 70% of surgical site infections (SSIs) are detected after discharge, while the median hospital length of stay for surgical patients is decreasing at a faster rate than before. Hospitals applying this efficient strategy have reported a 32% decrease surgical site infection and 48% drop in complications [24,25]. One infection control nurse per 250 beds, the training of an infection control physician, and an approach for notifying practicing surgeons of infection rates are another few programs that have been attempted but have been shown to be ineffective in hospitals in developing countries [26].

Limited studies have investigated the incidence and predictors of surgical site infection including American Society of Anesthesiologists score, postoperative hemoglobin levels, the presence of meconium, postpartum hemorrhage, and antibiotic prophylaxis post CS using a cohort study design in Ethiopia [10]. This study aimed to determine the incidence and predictors of surgical site infection post-CS among women who gave birth in public hospitals in the study area. This study will provide valuable evidence for governmental and non-governmental organizations working in the field of healthcare-associated infections by offering current data on the incidence and predictors of surgical site infections. Additionally, the findings will assist healthcare professionals, hospital administrators, program managers, and researchers in developing and implementing effective strategies to reduce SSI incidence, control contributing factors, and prevent related complications.

## Methods

### Study area and period

The study was conducted in the public hospitals of the East Wollega Zone, an administrative zone in the Oromia regional state in the western part of Ethiopia. Nekemte City, the capital of East Wollega Zone, is located 331 km from Addis Ababa, the capital city of Ethiopia. The zone comprises five public hospitals, 58 health centers, and 287 health posts. Among selected hospitals for this study, Wollega University Comprehensive Specialized Hospital and Nekemte Comprehensive Specialized Hospital, are located in Nekemte City, while Arjo Primary Hospital is located 49 km from Nekemte City. The study was conducted from January 15, 2024, to April 14, 2024.

### Study design

A hospital-based prospective cohort study was conducted at East Wollega Public hospitals.

### Source and study population

The source population consisted of all mothers who underwent cesarean deliveries at public hospitals in East Wollega. The study population included all mothers who underwent cesarean deliveries at the three selected East Wollega Public Hospitals during the study period.

### Eligibility criteria

All mothers who underwent cesarean deliveries at selected hospitals during the study period and agreed to participate were included in the study. Mothers who returned for follow-up or could be contacted via personal or attendant telephone were also included. However, mothers who delivered by cesarean section and died within 24 hours postoperative were excluded. Additionally, mothers diagnosed with uterine rupture, those on therapeutic antibiotics, or those without a personal or attendant telephone were also excluded from the study.

### Sample size determination and sampling technique

The sample size was calculated using the Schoenfeld formula for common predictors, such as multiple vaginal examinations, lack of antenatal care (ANC) follow-up, type of incision, and operations performed by an intern or junior doctor. These factors were found to be strongly associated with surgical site infections (SSI) among mothers who underwent cesarean delivery, according to studies conducted in various regions [6,10].

The total sample size was calculated as n = $\frac{E}{Pr(E)}$ [27]

Where n = desired sample size, E = the number of events that interested and Pr (E) =the probability that the event of interest


$$\text{E}=\frac{{\left(\frac{Z\alpha }{2}+Z\beta \right)}^{2}}{\left(lnHR{)}^{2}P(1-p\right)}$$

Zα/2 represents the critical value or normal distribution at 95% CI, which equals 1.96 (Z value at α = 0.05). Zβ = the power of the study 0.84 HR = hazard ratio Pr (E) =SSI rate who underwent Cesarean Delivery. As a result, it is recommended to use the largest sample size derived from all calculations to meet all study objectives. Therefore, a total of 408 mothers who underwent cesarean delivery (CD) were needed for the study. Participants were enrolled serially at each hospital until the target sample size was achieved.

### Sampling technique

Using purposive sampling technique, Three hospitals were selected from the five public hospitals in East Wollega. Cesarean delivery reports from the last three months of these hospitals determined the sample size for each facility: 407 from Wollega University Comprehensive Specialized Hospital, 300 from Nekemte Comprehensive Specialized Hospital, and 60 from Arjo Primary Hospital. The total source population over three months were 407 + 300 + 60 = 767407 + 300 + 60 = 767. Therefore, the sample sizes were calculated as follows:

For Wollega University Comprehensive Specialized Hospital: 407×408/767=216

For Nekemte Comprehensive Specialized Hospital: 300×408/767=160

For Arjo Primary Hospital: 60×408/767=32

**Fig 1:**
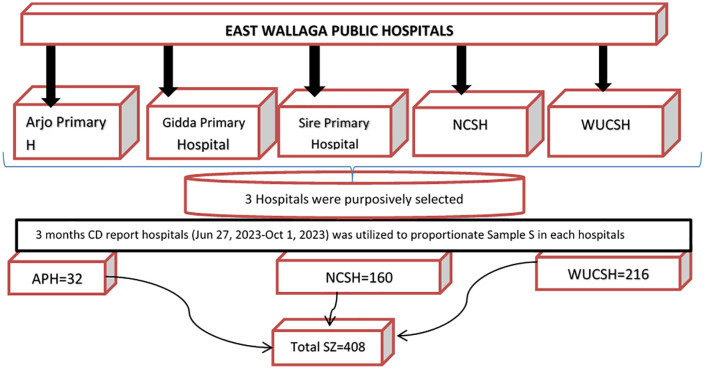
Schematic presentation of sampling procedure To determine predictors of surgical site infections following cesarean delivery in public hospitals of East Wollega, Ethiopia, 2024. Then a unique study ID number was given immediately after their operation.

A consecutive sampling technique was then employed, in which every post-operative mother who met the inclusion criteria for cesarean delivery was serially enrolled until the required sample size was achieved “Fig 1”.

### Data collection procedure

A validated data collection instrument has been derived from the ‘WHO Protocol for Surgical Site Infection Surveillance with a Focus on Settings with Limited Resources’ and customized to meet local needs [28]. Data was collected from all mothers who underwent Cesarean Delivery in selected hospitals during the study period who met the inclusion criteria through face-to-face interviews, medical record reviews, a telephone interview, and direct observations of the clients’ wounds using the data collection instrument. Data was collected by a resident/ General Practitioner and a nurse from each hospital.

### Follow up period

Data was collected during the hospital stay using the WHO Surgical Site Infection (SSI) Surveillance Checklist. The ward healthcare practitioner examined the wound daily and checked each participant for any signs and symptoms of SSI during their hospital stay. Participants were followed for thirty days to detect SSI, in accordance with the WHO Protocol for Surgical Site Infection Surveillance. Upon discharge, all participants were asked to provide one or more phone numbers, either their own or those of their attendants, for post-discharge communication. All participants and their attendants were informed about the signs and symptoms of surgical site infection (SSI). Clear instructions and orientation were provided to help them actively report any signs or symptoms of SSI. They were instructed to inspect the incision site at least twice a day for any indications of infection and to take notes on the onset of SSI signs and symptoms.

In addition to scheduled phone call interviews using the WHO checklist, a format was provided for participants that included the day, time, and phone number of the data collectors. Participants and their attendants who were unable to read and write were actively instructed to use this format to indicate the day and time of symptom onset and to inform the data collectors via phone call.

All participants were scheduled to return for follow-up after one week. On the day of the appointment, the resident physician or general practitioner took a brief history to determine whether the participant had developed symptoms of a surgical site infection (SSI) and had received treatment prior to the appointment date. Participants who did not attend their appointments were contacted by telephone to ascertain whether they had any signs or symptoms of SSI.

Participants were interviewed via phone call at least four times (at the end of weeks 1, 2, 3, and 4) after discharge. Those who developed surgical site infections (SSI) were identified through these telephone interviews. Participants who were able to visit the selected hospitals were engaged directly, while those unable to attend were referred to the nearest healthcare facility. A physical examination was conducted to determine whether the Participant was SSI positive or negative.

Additionally, further treatment was provided, and data collectors communicated with the healthcare providers at the facility to identify the results. After five unsuccessful phone calls during the follow-up period, if a participant’s phone was non-functional for three consecutive days of attempts, or if a participant refused to participate during the follow-up, they were considered as lost to follow-up.

### Data processing and analysis

Data entry was performed using Epi-Data version 4.6 and subsequently exported to STATA version 14 for further data cleaning and analysis. The incidence density (rate) of surgical site infections (SSI) was calculated based on overall person-time. Descriptive statistics of the data were summarized using frequency measurements and cross-tabulations. Graphs and tables were utilized to present the data effectively. The probability of surgical site infection failure was estimated using the Kaplan-Meier (KM) method, along with the log-rank test. The KM method was also employed to compare survival times between groups of categorical variables. A multicollinearity test was performed to check for collinearity between independent variables. The variance inflation factor (VIF) was assessed to determine whether it was greater than or less than ten. In this study, the mean VIF was 1.39, which falls within an acceptable range.

**Fig 2:**
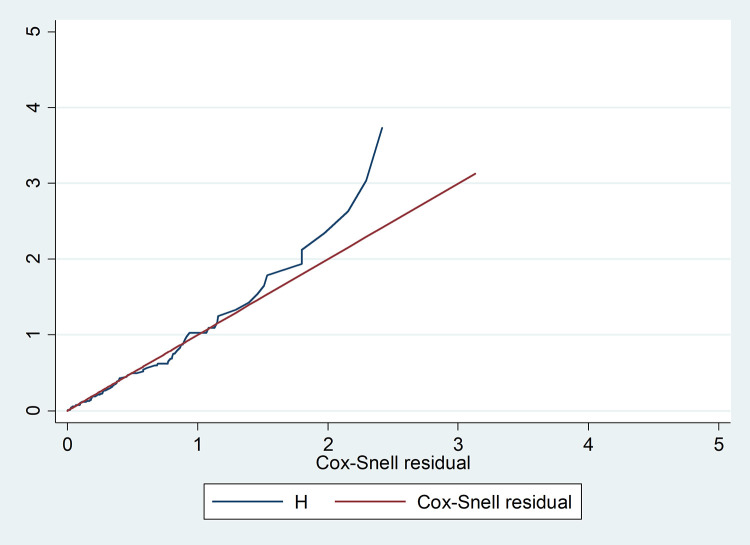
Cox-snell Nelson -Alen cumulative hazard graph on mothers underwent cesarean Delivery in East Wollega Public Hospitals between January 15/2024 to April 14/2024 (n = 408). The multi-variable analysis with a Cox proportional hazard model was made. Finally, variables with a P ≤ 0.05 in multi-variable analysis were declared as predictors of SSI.

Bi-variable analysis was analyzed and variables those P values less than or equal to 0.25 were included in the multi-variable survival models. The cox proportional hazard (PH) assumptions was checked using by both Schoenfeld residuals statistics (global test = 0.1432) and in the graphical method the curve of different group were parallel to each other. All variables fulfilled this PHA assumption (p-value > 0.05). Also the Cox Snell residual test was used to check the final model’s adequacy. The hazard function was nearly follows the 45° line but begins to deviate as the number of censored patients increases, showing that residuals have a standard censored exponential distribution with the hazard ratio. Therefore, the model is considered a good fit for the data “Fig 2”.

### Data quality assurance

Data collectors received short training on data collection instruments and procedures. The data collection tools were pretested on twenty-one participants (5%) at Sibu Sire Primary Hospital to ensure reliability and consistency before the actual data collection began. This pretesting aimed to identify any missing components or challenges during implementation. Validation of data collection tools was checked by advisors. At end of data collection for each participant, the investigator reviewed the postoperative SSI assessment checklist to ensure its completeness. When an error or omission was found, the investigator promptly was communicate the data collectors to address the situation.

### Operation definition

**Surgical site Infection (SSI)-** infection occur within thirty days of the surgical operation at the incision site and/or deeper underling tissue spaces and organs [29].

**Event**: was the first surgical site infection occur after a cesarean delivery procedure.

**Start time**: was post-operative day one (the procedure day)

**End time:** was thirty post-operative day.

**Survival time**: Time (in days) occurred between the end of the surgery and the development of surgical site infection after cesarean delivery.

**Censored**: Participant who died during the follow-up without developing SSI, or who did not develop SSI until thirty postoperative days or; non-response participant (after five calls); participant whose phone did not work for three consecutive days of trial; and patients who refused to participate during the follow-up.

**Body Mass Index (BMI):** is a medical screening tool that divides a person’s weight in kilograms by their height in meters squared. For this study, utilize the following CDC guidelines for adult BMI classification: 18.5 = underweight, 18.5 to 24.9 = a normal weight, 25.0 to 29.9 = overweight, and 30 and higher = obesity [30].

**Mid-upper arm circumference (MUAC):** is a measurement that enables medical professionals to quickly assess whether a patient is acutely malnourished. For this study <22 = malnutrition, ≥ 22 = Normal [30].

**Premigravda:** Premigravida (PG), defined as a woman who conceives for the first time [31].

### Wound Class

- **Clean**: Cesarean section wounds in cases where the procedure is done elective, no pre-rupture of membranes or trial of labour
- **Clean Contaminated**: Cesarean section wounds in cases where the procedure is performed under controlled and planned circumstances, and entry into the genital tract occurs without unusual contamination.
- **Contaminated**: Cesarean section wounds where the procedure is performed in the background of prolonged rupture of membranes, chorioamnionitis, obstructed labor, or an existing clinical infection.

### Type of SSI

- **Superficial surgical site infection (SSSI)**: an infection that occurs within 30 days after an operation and is limited exclusively to the skin and subcutaneous tissue of the incision.
- **Deep surgical site infection**: an infection that occurs within 30 days after the operation affects the deep soft tissues of the incision, specifically the fascial and muscle layers.
- **Organ/Space surgical site infection**: involves any part of the anatomy, such as an organ or a body cavity (space between organs), other than the skin incision, fascia, or muscle layers.

### Ethical considerations

Wollega University’s Institute of Health Science Research ethical review committee was provide ethical clearance. A letter was issued under Reference No. WU/RD/735 by Wollega University’s Research Ethical Review Committee on January 3, 2024. Also support letter was written for each hospital from institute of health sciences and a permission letter was obtained from the selected East Wollega Public hospitals. Before collecting individual data, participants’ written consent was obtained. Data collectors read the consent form to those who were unable to read. For those who could read, they provided the consent form directly. Afterward, the data collectors explained the title, purpose, and societal benefits of the study. Once the participants understood, they signed the consent form, and data collection proceeded. The participants’ data was accessible only to the data collectors, while the final summarized data was exclusively available to the investigator and advisors. All information was presented without disclosing the participants’ names.

## Results

This multi-center prospective cohort study was conducted from January 15, 2024, to April 14, 2024, at three public hospitals in East Wollega: Wollega University’s Comprehensive Specialized Hospital, Nekemte Comprehensive Specialized Hospital, and Arjo Primary Hospital. A total of 408 mothers who underwent cesarean delivery were included in the study and followed for thirty days post-delivery to detect surgical site infections (SSI). Out these, 343 participants (84.07%) completed their follow-up. Approximately 61 participants (14.95%) were lost to follow-up, and 4 participants (0.98%) were died during the study period.

**Fig 3:**
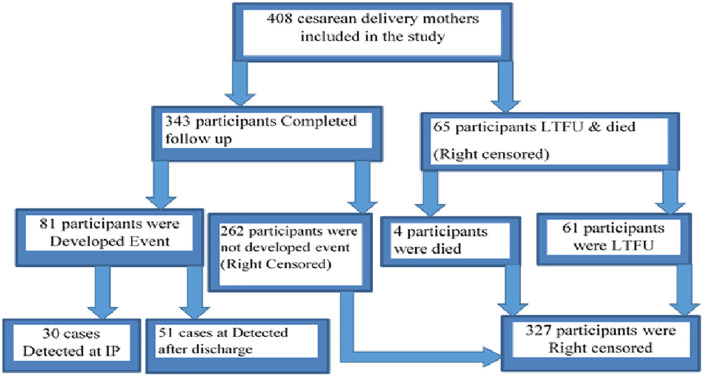
Overall recruitment and follow-up process of Cesarean delivery mother in East Wollega public Hospitals, Ethiopia, January 15–April 14/2024.

Among the cases detected, around 30 (37.04%) occurred during hospital stays or prior to discharge, while the majority, 51 cases (62.96%), were identified after participants were discharged from the hospitals. Most cases (21%) were detected on the seventh postoperative day, with a median time to event of 7 days (95% CI: 6–9) “Fig 3”.

### Socio demographic characteristics and behavioral related factors

The mean age of the participants included in this study was 26 years (95%: CI 15, 38) with standard deviation of 0.219. Almost 400 (98.04%) participants were married. More than half of the participants, 224 (54.90%), were came from rural areas. Most of the 373 (91.42%) participants were Oromo ethnic groups. About 128 (31.37%) of the participants completed secondary school, 106(25.98%), and 75 (18.38%) attended primary school, and they can read and write, respectively. More than three-fourths of the participants, 314 (76.96%), follows the protestant religion. The majority of participants, 261 (63.97%), were housewives, followed by government employees at 61 (14.95%). The remaining participants included 46 (11.27%) farmers and 43 (10.54%) in other occupations. About 19 (4.66%) participants consumed alcohol, and 11 (57.89%) reported consuming alcohol regularly. Very few 15 (3.68%) and 3 (0.74%) participants were smokers and were chat chewers, respectively “Table 1”.

**Table 1:** Socio-demographic characteristic and Behavioral related factors of participants among mothers underwent cesarean delivery in East Wollega Public Hospitals between January 15/2024 to April 14/2024 (n = 408).

Variables	Category	SSI		Frequency (%)
		Event (%)	Censored (%)	
Age	<24	22(27.16)	112(34.25)	134(32.84)
	24-34	55(67.90)	200(61.16)	255(62.50)
	≥35	4(4.94)	15(4.59)	19(4.66)
	Mean	Std. Dev.	Min	Max
	26.47	4.44	15	38
Marital status	Married	79(97.3)	321(98.17)	400(98.04)
	Divorced	1(1.23)	0(0.0)	1(0.25)
	Widowed	1(1.23)	6(1.83)	7(1.72)
Education Level	Cannot read & write	12 (14.81)	18(5.50)	30 (7.35)
	Can read & write	19 (23.46)	56(17.13)	75 (18.38)
	Primary school (2–71 –8 )	13 (16.05)	93(28.44)	106(25.98)
	Secondary school (10,9 –12 )	24 (29.63)	104(31.80)	128 (31.37)
	College/university and above	13(16.05)	56(17.13)	69(16.91)
Place of Residence	Rural	44(54.32)	180(55)	224(54.90)
	Urban	37(45.95)	147(45)	184(45.10)
Ethnicity	Oromo	73(90.2)	300(91.7)	373(91.42)
	Amhara	8(9.8)	27(8.3)	35(8.58)
Religion	Protestant	57(70.4)	257(78.6)	314(76.96)
	Orthodox	14(17.3)	57(17.4)	71(17.40)
	Muslim	10(12.3)	13(4)	23(5.64)
Occupation	Farmer	12(14.81)	31(9.5)	43(10.54)
	House wife	50(61.73)	211(64.53)	261(63.97)
	Government employee	10(12.3)	51(15.6)	61(14.95)
	Other	9(11.11)	34(10.40)	43(10.54)
Smoking status	Nonsmoker	78(96.30)	315(96.33)	393(96.32)
	Smoker	3(3.70)	12(3.67)	15(3.68)
Alcohol consuming status	Yes	3(3.70)	16(4.89)	19(4.66)
	No	78(96.30)	311(95.11)	389(95.34)
alcohol consumption Pattern	Regularly	2(66.67)	9(56.25)	11(57.89)
	occasionally	1(33.33)	7(43.75)	8(42.11)
Chat chewing	Yes	1(1.23)	2(0.61)	3(0.74)
	No	80(98.77)	325(99.39)	405(99.26)

Other: students and merchants

### Medical related factors

Only 68(16.67%) participants were diagnosed with comorbidity. No adequate representatives’ participants were observed for HIV/AIDS 16(3.92%) during data collection. Almost 384 (94.12%) participants had a normal body weight, followed by 9 (2.21%), 15 (3.68) underweight and overweight, respectively. Most 396 (97.06%) participants had normal MUAC. About 336 (82.3%) and 72 (17.65%) participants were diagnosed with ASA class < III and ≥ III, respectively “Table 2”.

**Table 2:** Frequency distribution of medical related factors among mothers underwent cesarean Delivery in East Wollega Public Hospitals between January 15/2024 to April 14/2024 (n = 408).

Variables	Category	SSI		Frequency (%)
		Event (%)	Censored (%)	
Comorbidity	Yes	10(12.35)	58(17.74)	68(16.67)
	No	71(87.65)	269(82.26)	340(83.33)
Body mass index	Underweight	3(3.70)	6(1.83)	9(2.21)
	Normal weight	74(91.76)	310(94.80)	384(94.12)
	Overweight	4(4.94)	11(3.36)	15(3.68)
Mid–upper Arm circumference	Undernutrition	4(4.94)	8(2.45)	12(2.94)
	Normal	77(95.06)	319(97.55)	396(97.06)
HIV status	Reactive	2(2.47)	14(4.28)	16(3.92)
	Non-reactive	79(97.53)	313(95.72)	392(96.08)
client on ART	Yes	1(50.00)	6(42.86)	7(43.75)
	No	1(50.00)	8(57.14)	9(56.25)
Thickness of subcutaneous tissue	≤2 cm	72(88.89)	294(89.91)	366(89.71)
	>2 cm	9(11.11)	33(10.09)	42(10.29)
ASA class	<III	29(35.80)	307(93.88)	336 (82.35)
	≥III	52(64.20)	20(6.12)	72(17.65)

### Pregnant related factors

Majority 364 (89.22%) of participants had antenatal care (ANC) follow-up, and most of them, 177 (48.49%), started at 13–29 weeks, and 78 (21.37%) of participants started after ≥29 weeks. Two-thirds of participants, 242 (66.48%), had two to five contacts at health facilities for ANC. The majority of 279 (68.38%) participants were multi-paras, followed by 118 (28.92%), 11 (2.70%), Premiparous and grand multi-paras, respectively. About 115 (28.19%) participants had a previous cesarean history; out of them, 63 (55.26%) and 51 (44.74%) had one and two cesarean scars, respectively. More than half of the procedures, 262 (64.22%), were conducted between 38 and 40 weeks, followed by 127 (31.13%) for those ≥40 weeks, and 19 (4.66%) for <37 weeks. For the majority of participants, 285 (69.85%), partographs were not used during labor. Additionally, more than one-third of participants, 174 (42.65%), were not in labor during the procedure, while the remaining 192 (47.06%) included 42 (10.29%) mothers who were in labor for <24 hours and ≥24 hours, respectively. Per vaginal examination was done for most participants, 245 (60.05%). Among these, 220 (53.92%) underwent the examination 1–5 times, while 25 (6.13%) had it done six times. More than half 231(56.62%) of the membrane was ruptured during surgery. About 84(36.36%) of them ruptured before 24 hours. About 16 (3.92%) and 62(15.20%) participants were diagnosed with chorioaminitis and grade three meconium, respectively. Limited numbers of participants 32 (7.84) were developed postpartum hemorrhage during and after surgery “Table 3”

**Table 3:** Frequency distribution of Pregnant related factors among mothers underwent cesarean Delivery in East Wollega Public Hospitals between January 15/2024 to April 14/2024 (n = 408).

Variables	Category	SSI		Frequency (%)
		Event (%)	Censored (%)	
Antenatal care follows up	Yes	66(81.48)	298(91.13)	364(89.22)
	No	15(18.52)	29(8.87)	44(10.78)
ANC booking start at how many weeks	≤12weeks	19 (28.79)	91 (30.43)	110 (30.14)
	13–29 weeks	29 (43.94)	148 (49.50)	177 (48.49)
	≥29 weeks	18 (27.27)	60 (20.07)	78 (21.37)
Number of ANC contact	1 time	16 (24.24)	36(12.08)	52 (14.29)
	2–5 times	36 (54.55)	206(69.13)	242 (66.48)
	≥6 times	14 (21.21)	56(18.79)	70 (19.18)
Parity	Premiparous	21(25.93)	97(29.66)	118(28.92)
	Multipara	54(66.67)	255(68.81)	279(68.38)
	Grand multipara	6(7.41)	5(1.53)	11(2.70)
Previous cesarean delivery	Yes	21(25.93)	95(28.75)	116(28.19)
	No	60(74.07)	233(71.25)	293 (71.8)
Number of cesarean delivery	1 time	13(61.90)	50(53.76)	63(55.26)
	2 times	8(38.10)	43(46.24)	51(44.74)
Gestational age at cesarean delivery	<37 weeks	6(7.41)	13(3.98)	19(4.66)
	38–40 weeks	53(65.43)	209(63.91)	262(64.22)
	≥40weeks	22(27.16)	105(32.11)	127(31.13)
Partograph use	Yes	23 (28.40)	100 (30.58)	123 (30.15)
	No	58 (71.60)	227 (69.42)	285 (69.85)
Mother has been in labor before operation	No labor	39 (48.15)	135 (41.28)	174 (42.65)
	<24hrs	24 (29.63)	168 (51.38)	192 (47.06)
	≥24hrs	18 (22.22)	24 (7.34)	42 (10.29)
per Vaginal Examination done	No exam	27(33.33)	136(41.59)	163(39.95)
	1–5 times	44(54.32)	176(53.82)	220(53.92)
	≥6 times	10(12.35)	15(4.59)	25(6.13)
Membrane state	Ruptured	50(61.73)	181(55.35)	231(56.62)
	Intact	31(38.27)	146(44.65)	177(43.38)
duration of membrane rupture	<24hrs	29(58.00)	118(65.19)	147(63.64)
	≥24hrs	21(42.00)	63(34.81)	84(36.36)
Chorioaminonitis	Yes	8(9.88)	8(2.45)	16 (3.92)
	No	73 (90.12)	319(97.55)	392 (96.08)
Meconium	Yes	44(54.32)	18(5.50)	62(15.20)
	No	37(45.68)	309(94.50)	346(84.80)
Post-partum hemorrhage	Yes	22(27.16)	10(3.06)	32 (7.84)
	No	59(72.84)	317(96.94)	376 (92.16)

### Procedural related factors

Most participants, 277(67.89%), were operated as emergency. Most participants 261 (63.97%) were stayed in the hospital less than twenty-four hours before the procedure. Most procedures, 376 (92.16%), were performed at tertiary hospitals. The surgical site was cleaned with iodine, alcohol, and chlorohexidine (163 (39.95%), 216 (52.94%), and 29 (7.11%), respectively. Skin was incised transversely and mid-line 361 (88.48%) and 47 (11.52%), respectively. Nearly three-fourths of procedures (295 (72.30%) took more than thirty-eight minutes, and 113 (27.70%) were completed within thirty-eight minutes. More than one-third of the procedures, 157 (38.48%), were performed by residents with more than three years of experience, followed by 151 (37.01%) performed by gynecologists and 100 (24.51%) by integrated emergency and surgical officers, respectively. Most procedures, 380 (93.14%), were closed continuously followed by 28 (6.86%) interrupted skin closure techniques. Most of the 321(78.68%) participants were diagnosed with ≥11 g/dl post-operative hemoglobin test, followed by 87(21.32%) with <11 g/dl post-operative hemoglobin. For the majority of participants, 289 (70.83%), antibiotic prophylaxis was administered, while it was not provided for 119 (29.17%) participants. Among the 289 who received prophylaxis, 81 (28.03%) took antibiotics within thirty minutes before surgery, and 208 (71.97%) took them more than thirty minutes prior. Post-surgery antibiotics were given to 167 (40.93%) participants, while they were not ordered for the remaining 167 (40.93%). Of those who received antibiotics, 17 (10.18%) took them for only twenty-four hours, whereas 150 (89.82%) took them for more than twenty-four hours. Additionally, only 28 (6.86%) participants received a blood transfusion. Majority (291 (71.32%) of participants were operated under spinal anesthesia, followed by 117 (28.68%) under general anesthesia “Table 4”.

**Table 4:** Frequency distribution of Procedural and medication related factors among mothers underwent cesarean delivery in East Wollega Public Hospitals between January 15/2024 to April 14/2024 (n = 408).

Variables	Category	SSI		Frequency (%)
		Event (%)	Censored (%)	
days after admission when procedure was done	≤24hrs	53(65.43)	208(63.61)	261(63.97)
	>24hrs	28(34.57)	119(36.39)	147(36.03)
where the procedure performed	Tertiary hospital	73(90.12)	303(92.66)	376(92.16)
	Primary hospital	8(9.88)	24(7.34)	32(7.84)
Skin preparation	Iodine	32 (39.51)	131 (40.06)	163 (39.95)
	Alcohol	39 (48.15)	177 (54.13)	216 (52.94)
	Chlorohexidine	10 (12.35)	19 (5.81)	29 (7.11)
Surgical site Hair removal	Yes	63(77.78)	259(79.20)	322(78.92)
	No	18(22.22)	68(20.80)	86(21.08)
Type of skin incision	transverse	69(85.19)	292(89.30)	361(88.48)
	Midline	12(14.81)	35(10.70)	47(11.52)
Duration of surgery	≤38 minutes	21(25.93)	92(28.13)	113(27.70)
	>38 minutes	60(74.07)	235(71.87)	295 (72.30)
operation is performed	IESO	18(22.22)	82(25.08)	100(24.51)
	Resident ≥3 Years	35(43.21)	122(37.31)	157(38.48)
	Gynecologist	28(34.57)	123(37.61)	151(37.01)
Skin closure technique	Interrupted sutures	8(9.88)	20(6.12)	28(6.86)
	Continuous	73(90.12)	307(93.88)	380(93.14)
Skin closed by	Absorbable	76(93.83)	315(96.33)	391(95.83)
	Non absorbable	5(6.17)	12(3.67)	17(4.17)
Post-operative hemoglobin	<11g/dl	49(60.49)	38(11.62)	87(21.32)
	≥11g/dl	32(39.51)	289(88.38)	321(78.68)
Antibiotic prophylactic given	Yes	25(30.86)	264(80.73)	289(70.83)
	No	56(69.14)	63(19.27)	119(29.17)
time of antibiotic prophylactic given before skin incision	≤30 minutes	9 (36.00)	72(27.27)	81 (28.03)
	>30 minutes	16 (64.00)	192(72.73)	208 (71.97)
Use of antibiotic post-surgery	Yes	38(46.91)	129(39.45)	167(40.93)
	No	43(53.09)	198(60.55)	241(59.07)
Post-operative antibiotic use duration	≤24hours	7(18.42)	10(7.75)	17(10.18)
	>24 hours	31(81.58)	119(92.25)	150(89.82)
Blood transfusions	Yes	19(23.46)	9(2.75)	28(6.86)
	No	62(76.54)	318(97.25)	380(93.14)
Type of anesthesia	General	47(58.02)	70(21.41)	117(28.68)
	Spinal	34(41.98)	257(78.59)	291(71.32)

#### Indication.

**Fig 4:**
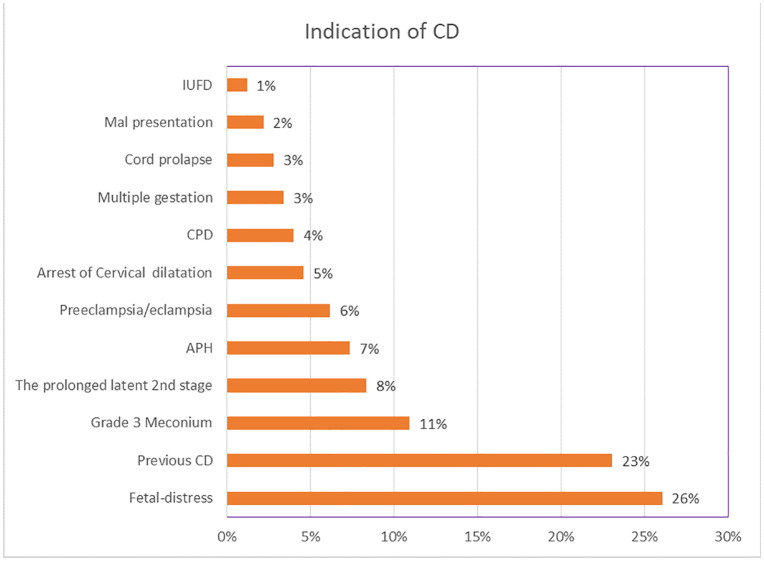
Indication of cesarean delivery in East Wollega Public Hospitals between January 15/2024 to April 14/2024 (n = 408).

Some participants have multiple indications for cesarean delivery. The most common indication of cesarean delivery was fetal distress (131, 26%), followed by previous cesarean delivery (116, 23%) and grade 3 meconium-stained amniotic fluid (55, 11%). prolonged latent second stage of labor 41(8%), and Antepartum hemorrhage 36 (7%), and were also indication of cesarean delivery “Fig 4”.

**Fig 5:**
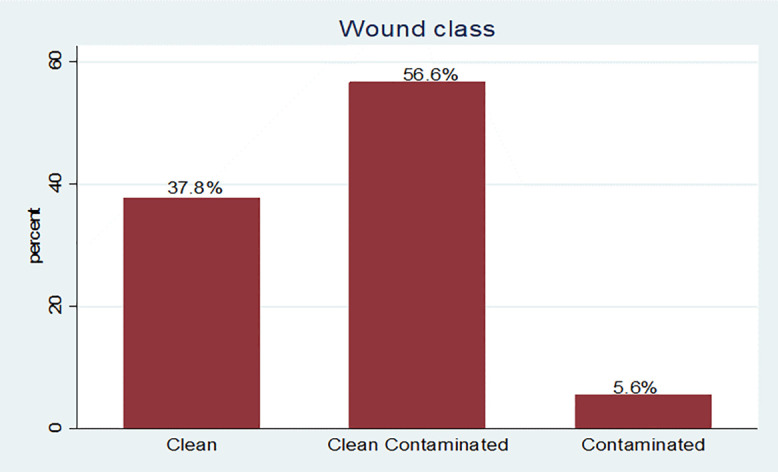
Frequency of wound class at East Wollega Public Hospitals between January 15/2024 to April 14/2024. Most 277 (67.89%) cases were operated as emergency “Fig 6”.

The majority of wound classes were 231(56.62%) clean contaminated, followed by 154 (37.5%) and 23 (5.64%) clean and contaminated, respectively “Fig 5”.

**Fig 6:**
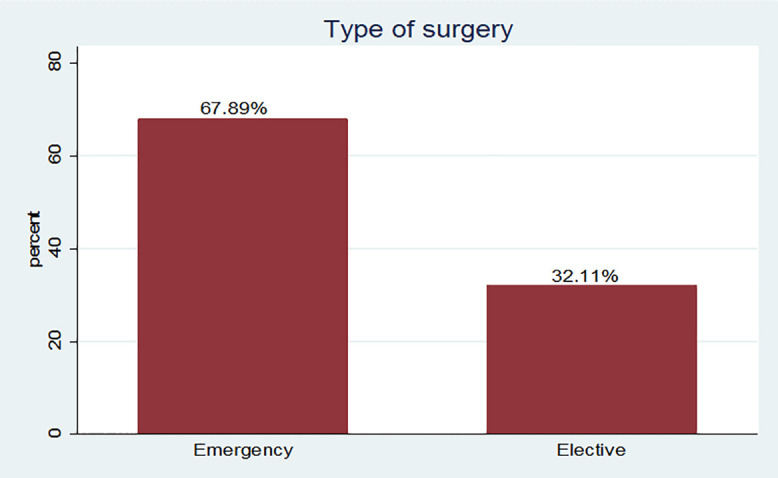
Frequency distribution of type of surgery among mothers underwent cesarean Delivery in East Wollega Public Hospitals between January 15/2024 to April 14/2024 (n = 408).

**Fig 7:**
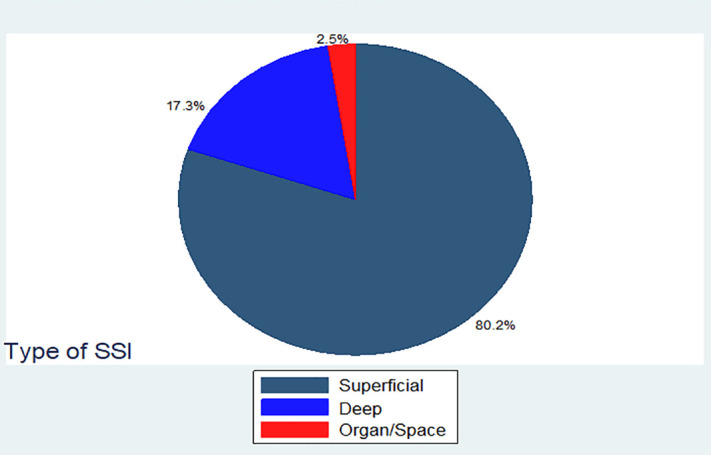
Frequency distribution of type of SSI among mothers underwent cesarean delivery in East Wollega Public Hospitals between January 15/2024 to April 14/2024 (n = 81). Most participants 327(80.15%) were censored and 81(19.85%) were event “Fig 8”.

More than third and fourth 65 (80.3%) of surgical site infections were superficial, while the rest were deep and organ/space 14 (17.3%) and 2 (2.5%), respectively “Fig 7”.

**Fig 8:**
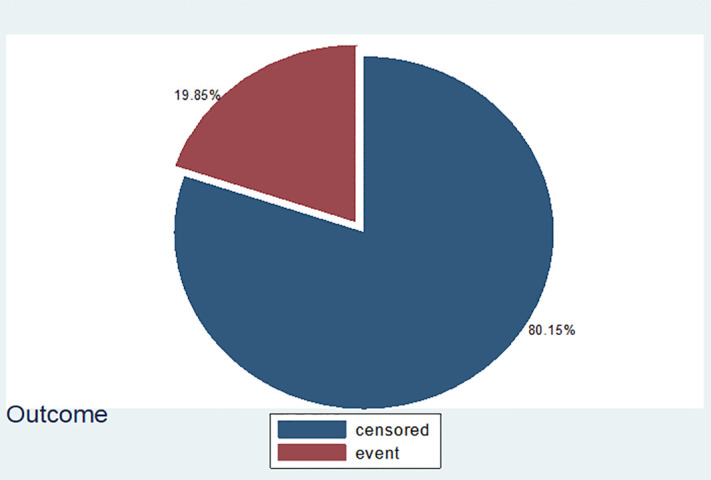
Frequency distribution of follow up outcome of SSI among mothers underwent cesarean delivery in East Wollega Public Hospitals.

**Fig 9:**
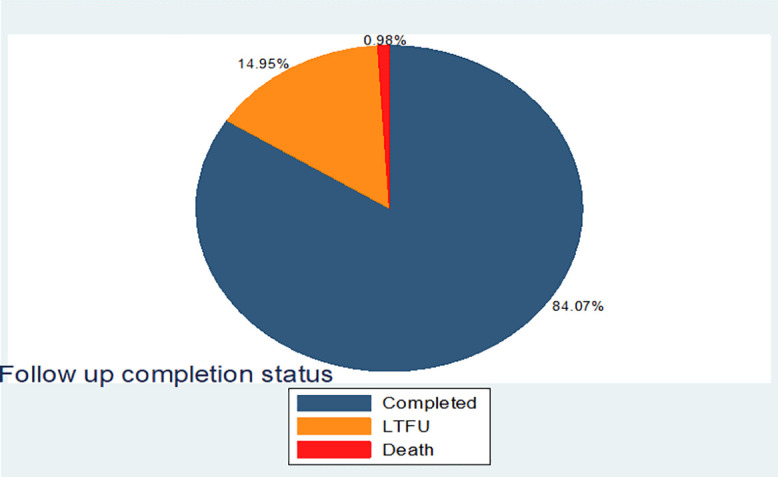
Frequency distribution of follow up completion status of participants among mothers underwent cesarean Delivery in East Wollega Public Hospitals between January 15/2024 to April 14/2024 (n = 408).

Most participants 343(84.07%) completed their follow-up, while the rest were lost to follow-up and death 61(14.95%) and 4(0.98%), respectively “Fig 9”.

### Incidence rate

Out of 408 participants, eighty one developed a surgical site infection. 65(80.25%) were superficial, followed by 14 (17.28%) and 2 (2.47%) deep and organ/space surgical site infections, respectively. Total person time observation was 8662 days, and it yielded 9.4 cases per 1,000 person time observation with a confidence interval of (95% CI: 7.5–11.6).

#### Survival time analysis.

A life table and survival plot were used to determine the probability of survival or not acquiring a surgical site infection, respectively. Because of the high censoring rate in this cohort, the overall median survival time could not be calculated. This study showed that the cumulative probability of not acquiring a SSI at the end of the second postoperative day was 100%. At the end of the third POD, the probability of survival was 99.2% (95% confidence interval: 0.97–0.9974), with a standard error of 0.0046. At the end of the 15^th^ POD of stay, the probability of survival was found to be 76.7% (95% CI: 0.7193–0.8084) with a standard error of 0.0227. No SSI was detected after the end of the 16^th^ POD. 76.8% of participants survived without developing a SSI at the end of the 30-day follow-up study. The majority of 57 (70.3%) SSIs occurred between the 5^th^ and 10^th^ postoperative days, followed by 16 (19.8%) between the 11^th^ and 15^th^ POD. About 8 (9.9%) cases occurred between the 3^rd^ and end of the 4^th^ post-operative day follow-up. Most cases (21%) were detected on the seventh post-operative day. Median time for event is 7(95% CI: 7–9).

#### Kaplan-Meier survival curves.

**Fig 10:**
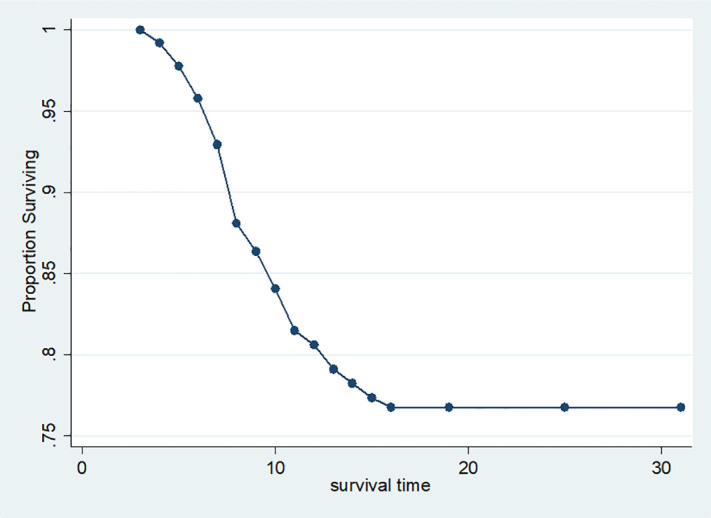
KM survival curve for comparison of survival time among mothers underwent cesarean Delivery in East Wollega Public Hospitals between January 15/2024 to April 14/2024 (n = 408).

The overall Kaplan-Meier estimate revealed that the probability of survival without SSI of the mother was maximum in the first two postoperative days but declined on the third day as follow-up duration rose. However, after 16^th^ PODs of follow-up, the graph showed no significant difference “Fig 10”.

**Fig 11:**
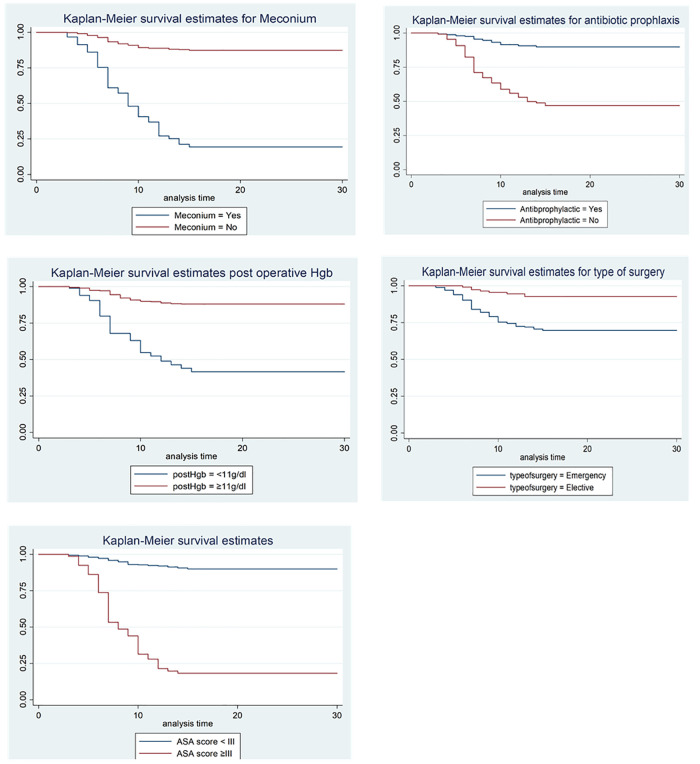
KM survival curve for comparison of survival time within categories of predictors to determine incidence and predictors of SSI among mothers underwent cesarean Delivery in East Wollega Public Hospitals between January 15/2024 to April 14/2024.

To test the equality of survival curves for different categorical variables, the Kaplan-Meier survival curves and log-rank test were tested. The test statistics that were gained from the test showed that there was a significant difference in the survival function (curve) of meconium (X^2^ for log-rank test = 155.79, P = 0.000), antibiotic prophylactic (X^2^ = 84.39, P = 0.000), post-operative hemoglobin (X^2^ = 348.49, P = 0.000), type of surgery (X^2^ = 21.53, P = 0.000), and ASA class (X^2^ = 44.20, P = 0.000) “Fig 11”.

### Predictors of surgical site infection

This study applied bi-variable Cox regression analysis to forty nine variables. Among them, seventeen of them, namely: age of participant, educational status, comorbidity, ASA score, post-partum hemorrhage, postoperative hemoglobin, antenatal care (ANC) follow-up, parity, vaginal examination, gestational age, meconium, chorioaminitis, type of surgery, surgical wound classification for cesarean section, antibiotic prophylactic, blood transfusions, and type of anesthesia used, were scored a p-value of less than or equal to 0.25. They were being considered for multi-variable analysis.

### Multivariable cox proportional regression model

In multi-variable cox proportional analysis, meconium, ASA score, post-operative hemoglobin, antibiotic prophylactic, and emergency surgery were predictors of surgical site infection after cesarean delivery pv ≤ 0.05 “Table 5”.

**Table 5:** Multi-variable in cox proportional analysis to identify Predictors of Surgical Site Infection among mothers underwent cesarean Delivery in East Wollega Public Hospitals between January 15/2024 to April 14/2024 (n = 408).

Predictors	Category	Status		CHR(95% CI)	AHR (95% CI)	P
		Event (%)	Censored (%)			
Age	<24	22(27.16)	112(34.25)	Ref	Ref	
	24-34	55(67.90)	200(61.16)	1.3(0.83-2.24)	1.4(0.76-2.8)	0.246
	≥35	4(4.94)	15(4.59)	1.34 (0.46-3.9)	2.8 (0.65-12)	0.164
Education Level	Cannot read & write	14 (17.28)	15(4.6)	2.5 (1.14-5.4)	0.3 (0.09-1.3)	0.118
	Can read & write	19 (23.46)	58(17.74)	1.4(0.72-2.9)	0.7 (0.3-1.9)	0.581
	Primary school	10 (12.35)	93(28.44)	0.68(0.3-1.4)	0.54 (0.24-1.3)	0.204
	Secondary school	21 (25.93)	105(32.11)	1.02(0.5-2)	0.6(0.3-1.38)	0.265
	College/university and above	17(20.99)	56(17.13)	Ref	Ref	
Comorbidity	Yes	50(61.73)	47(14.37)	4.4 (2.4-8.25)	2(0.88-5.2)	0.190
	No	31(38.27)	216(85.63)	Ref	Ref	
ASA class	<III	29(35.80)	307(93.88)	Ref	Ref	
	≥III	52(64.20)	20(6.12)	14.1(8.8-22.6)	5.3(2.8-9.78)	0.000***
Post-operative hemoglobin test	<11g/dl	49(60.49)	38(11.62)	6.3 (4-9.89)	1.78 (1.04-3)	0.035*
	≥11g/dl	32(39.51)	289(88.38)	Ref	Ref	
Antenatal care (ANC) follows up	Yes	66(81.48)	298(91.13)	Ref	Ref	
	No	15(18.52)	29(8.87)	2.41(0.37-4.2)	0.99 (0.48-2)	0.994
Parity	Premiparous	21(25.93)	97(29.66)	Ref	Ref	
	Multipara	54(66.67)	255(68.81)	1.09(0.6-1.8)	1.05(0.52-2)	0.881
	Grand multipara	6(7.41)	5(1.53)	3.4(1.4-8.66)	1.11 (0.26-4.7)	0.885
Gestational age	<37 weeks	6(7.41)	13(3.98)	2.2 (0.9-5.1)	1.5(0.52-4.5)	0.423
	38–40weeks	53(65.43)	209(63.91)	Ref	Ref	
	≥40weeks	22(27.16)	105(32.11)	0.7 (0.4-1.2)	1.4(0.75-2.7)	0.270
Vaginal Examination done	No exam	27(33.33)	136(41.59)	Ref	Ref	
	1–5 times	44(54.32)	176(53.82)	1.1(0.7-1.9)	0.5(0.25-1.05)	0.070
	≥6 times	10(12.35)	15(4.59)	2.78(1.3-5.7)	2.2(0.83-6.25)	0.107
Chorioaminitis	Yes	8(9.88)	8(2.45)	3.2(1.5-6.8)	0.5(0.16-1.77)	0.312
	No	73 (90.12)	319(97.55)	Ref	Ref	
Meconium	No	37(45.68)	309(94.50)	Ref	Ref	
	Yes	44(54.32)	18(5.50)	9.8 (6.3-15.42)	2.37 (1.3-4.12)	0.002**
Post-partum hemorrhage	Yes	22(27.16)	10(3.06)	4.5 (2.7-7.37)	0.8 (0.42-2)	0.614
	No	59(72.84)	317(96.94)	Ref	Ref	
Type of surgery	Emergency	73(90.12)	204(62.39)	4.7(2.2-9.76)	2.5(1.16-5.41)	0.019*
	Elective	8(9.88)	123(37.61)	Ref	Ref	
Surgical wound classification for cesarean section	Clean and Clean Contaminated	67(17.40)	318(82.60)	Ref	Ref	
	Contaminated	14(60.87)	9(39.13)	1.48 (0.56-3.9)	1.15(0.56-3.9)	0.777
Antibiotic prophylactic	Yes	25(30.86)	264(80.73)	Ref	Ref	
	No	56(69.14)	63(19.27)	6.6(4-10.7)	2.46(1.4-4.21)	0.001**
Blood transfusions	Yes	19(23.46)	9(2.75)	5.8 (3.4-9.7)	0.8 (0.4-1.85)	0.708
	No	62(76.54)	318(97.25)	Ref	Ref	
Type of anesthesia	General	47(58.02)	70(21.41)	3.5(2.3-5.59)	0.7(0.38-1.58)	0.487
	Spinal	34(41.98)	257(78.59)	Ref	Ref	

*** Very highly significant Pv ≤ 0.001 ** highly significant Pv ≤ 0.01 * Significant Pv ≤ 0.05

Participants who were diagnosed with meconium had 2.37 times hazards of acquiring SSI after cesarean delivery (AHR = 2.37, 95% CI: 1.3–4.12) when, compared to those that had no meconium. Participants who were diagnosed with a greater than or equal to class III ASA class had a 5.3 times risk of developing a SSI after cesarean delivery (ARH = 5.3, 95% CI: 2.8–9.78) when contrasted with those who were diagnosed with a less than class III ASA class. Participants who were diagnosed with less than or equal to eleven deciliters of postoperative hemoglobin had about two times higher hazards of acquiring surgical site infection after cesarean delivery (AHR = 1.78, 95% CI: 1.04–3.06) when contrasted with those who were diagnosed with greater than eleven deciliters of postoperative hemoglobin. Participants who were undergone cesarean delivery urgently had a 2.5 times higher risk of developing a surgical site infection after cesarean delivery (AHR = 2.5, 95% CI: 1.6–5.41) when contrasted with those operated on schedule. Participants who didn’t take antibiotic prophylaxis had 2.46 times hazards of acquiring surgical site infection after cesarean delivery (ARH = 2.46, 95% CI: 1.4–4.21) when contrasted with those who had taken antibiotic prophylaxis.

## Discussion

This prospective cohort study was aimed to determine the incidence and predictors of SSI after cesarean delivery. The incidence rate of this multi-center study finding is 9.4 per 1000 person/day (95% CI: 7.5, 11.6) and it identified different predictors of SSI, such as the presence of meconium, emergency surgery, the American Society of Anesthesiologists, post-operative hemoglobin, and antibiotic prophylaxis.

Regarding incidence density, “There is limited information available on the incidence density of surgical site infections (SSI) among mothers who undergo cesarean delivery. This study is consistent with a similar study conducted at Debre Markos Referral Hospital in Ethiopia [10]. The similarity might be due to the same study design and data collection method. The incidence density of the present study was lower than the previous reports from studies conducted at specialized hospitals in the Amhara region and Tunisia [8,9]. This discrepancy might be due to this study was include only cesarean delivery but above mentioned studies were include all of the general surgeries, which more cases were dirty and contaminated wounds. This Study finding is much higher than the studies conducted in Tanzania and Serbia’s [6,7]. This variation might be associated with differences in surgical techniques, patient care, medication, and hospital set-up.

Although surgical site infections (SSIs) typically develop within thirty days of surgery, identifying the exact timing of their onset is crucial for early detection, prevention, and intervention to mitigate their serious and potentially fatal complications. Understanding when an SSI occurs is also key to reducing the risk of wound related complications, such as bacteremia, from progressing to more severe conditions [32].

Due to the high censoring rate in this cohort study, the overall median survival time could not be estimated. 76.8% of participants survived without developing a SSI at the end of the 30-day follow-up study. Median time for event is 7(95% CI: 7–9). This finding was slightly in line with other studies done in Tanzania and Brazil. This similarity might be due to the use of the same data collection method and chance.

The findings of this study demonstrate a significant association between the presence of meconium and the development of surgical site infections (SSI). Participants diagnosed with meconium had a 2.3 times higher risk of acquiring an SSI following cesarean delivery compared to those without meconium. This result is consistent with a study conducted in Ethiopia [33]. Moreover, previous studies: A cohort of pregnant women complicated by premature labor underwent amniocenteses for culture the ammonitic fluid with meconium in it had a higher chance of having a positive culture than the ammonitic fluid without meconium [34,35]. The possible explanation is that contaminated incision site with tick meconium serves as good media for bacterial proliferation. Therefore close monitoring of labor is important to prevent meconium complications.

In this study, Participants who were undergone cesarean delivery urgently had 2.5 times higher risk of developing a SSI after cesarean delivery when contrasted with those operated on schedule. Similar findings have been reported by other studies [4,35–39]. This might be due to inadequate preoperative preparation, lack of proper control of other medical comorbidity, and higher risks for contamination in emergency surgeries. This finding could be attributable to the fact that, in emergency cases, membrane rupture and multiple vaginal examinations are more frequent. There is also an increased risk of bacterial contamination, breaks in sterile technique, and/or a lack of timely antibiotic prophylaxis.

Participants diagnosed with an ASA classification of III or higher had a 5.3 times greater risk of developing a surgical site infection (SSI) after cesarean delivery compared to those with an ASA classification lower than III. This is supported by a study conducted previously in Ethiopia, in India, in Uganda, in Rwanda and in USA [9,40–44]. This could be due to the patients those who are ASA class greater than III are unable to self-care, procedure is also preformed urgently for such like patients and other comorbidity can be the reason for the association of SSI and ASA class greater than III. On the other hand, a study done in Saudi Arabia [45] and Greece [46] reported that the ASA classification was insignificant. This disparity could be construed as the result of variations in the ASA class allocation process. Because the ASA definitions are based solely on the severity of the condition and do not take into account patient age, gender, weight, the type of procedure, the ability of the anesthetist or surgeon, or the degree of presurgical preparation, they are very subjective. As a result, both the size and the direction of the relationship between the SSI and ASA class may be significantly changed [45]. Therefore, anesthesiologists/anesthetist must take extreme caution when classifying special populations, such as pregnant women.

This study revealed that participants who did not receive antibiotic prophylaxis had a 2.5 times higher risk of acquiring a surgical site infection (SSI) after cesarean delivery compared to those who received antibiotic prophylaxis. This is consistent with previous findings in Ethiopia, in India and in Brazilian [37,47,48]. This might be because antibiotics have the ability to lower the bacterial population around the site of incision. Guidelines from the American Society of Hospital Pharmacists (ASHP) and other authors came to the conclusion that, with the aim of lowering the risk of infectious cesarean complications, using a single preventive dose—regardless of the kind of antibiotic was at least as effective as giving numerous doses [49]. In addition, when it regards preventing SSI following cesarean deliveries, Kelley Conroy and Errol Norwitz’s list of 10 evidence-based recommendations stated that “antibiotic prophylaxis significantly reduced infectious morbidity when it was given sixteen minutes before the skin incision, with no significant effect on neonatal outcome [50–52]. But, a study conducted in Kuwait argued that despite the use of prophylactic antibiotics, their patients developed SSI as contrasted with those who did not receive any antibiotics as prophylaxis [38]. Strict administration of pre-operative antibiotic prophylaxis is important to reduce the incidence of post-cesarean delivery SSI.

Participants with postoperative hemoglobin levels of 11g/dl or lower had about twice the risk of developing a surgical site infection (SSI) after cesarean delivery compared to those with hemoglobin levels above 11 g/dl.This finding consistent with the findings of other studies done in Ethiopia and in Thai-Myanmar [18,53–55]. This can be the result of decreased post-operative ambulation and hypo perfusion of the wound caused on by anemia. In general, a low hemoglobin content increases the risk of wound infection and inhibiting the tissues’ ability to get enough oxygen that limit macrophage activity and delay the healing process. Therefore Women who have post-operative hemoglobin less than or equal to eleven grams per deciliters are need close post-discharge follow-up for SSI.

### Limitation of study and strength of the study

The study’s estimated rate of surgical site infection may have been impacted by the incomplete follow-up of a few patients who were lost to follow-up. Due to hospitals lacked access to laboratory culture services, etiology of SSI couldn’t identified, including fungal or anaerobic bacterial infections. Nowadays post-surgery inpatient stay is gradually decreasing. However most SSI is developed after discharge, this study was capable of demonstrating the distribution of the SSI after discharge. It also enabled us to appreciate incidences of post-cesarean delivery SSI that might have been lost due to poor documentation if a retrospective cohort design was used.

## Conclusion and recommendation

This study aimed to determine the incidence and predictors of SSI after cesarean delivery at East Wollega public Hospitals. Incidence density of surgical site infection following cesarean delivery is relatively high in the study area. Most surgical site infections occurred between the seventh and ninth postoperative days. Various preventable factors, like pregnancy-related factors, procedural-related factors, medical-related factors, and medication-related factors, were observed in this study. The result indicate that the presence of meconium, emergency surgery, American Society of Anesthesiologists’ class ≥III, post-operative hemoglobin less than 11 g/dl, and absence of antibiotic prophylaxis were identified as predictors of SSI after cesarean section. The incidence rate of post-cesarean delivery SSI is relatively high in this study. It needs the organized effort of all stakeholder groups to reverse the situation. Therefore, the following recommendations are forwarded based on the results of the study: Close monitoring of labor is important to prevent meconium complications. Also strict administration of pre-operative antibiotic prophylaxis is help to reduce the incidence of post-cesarean delivery SSI. For emergence surgery, maintaining sterility techniques and providing pre-operative antibiotics may reduce SSI. Women who have an ASA class greater than or equal to III and post-operative hemoglobin less than or equal to eleven grams per deciliters are need close post-discharge follow-up for SSI. Implementation of a post-discharge SSI surveillance system is necessary to early identify and prevent complications of SSI because most of them occur after discharge. Hospitals should increase Public awareness about the risk factors of SSIs through health education to reduce their observed incidence density. Hospital administrators, infection prevention control staff, maternity ward head, and Operation Room head should be encouraged to enforce improved surgical techniques and facilitate the implementation of infection prevention protocols. Also Hospitals should improve their SSI surveillance systems as per WHO protocol to minimize the burden of SSI. Consistent personal and hand hygiene among all staff of the labor and delivery unit, Operation Room, and strict administration of pre-operative antibiotic prophylaxis could reduce the incidence of post-cesarean delivery surgical site infection.

## Supporting information

S1 File SSI data collection tools. (DOCX)

S2 File Tables. (DOCX)
