# Supplementary material for: Predictors of surgical site infections following cesarean delivery in public hospitals of West Ethiopia: A cohort study
Source: PLoS One. 2026 Jan 21;21(1):e0339930. doi: 10.1371/journal.pone.0339930 (PMC12822956; doi:10.1371/journal.pone.0339930)
Supplement: S1 File — (DOCX) [file pone.0339930.s001.docx]

**Unka odeeffannoo fi walii galtee**

Maqaan koo--------------------------------jedhama.Garee qorannoo mata-dureen isaa SABABOOTA FI **INFEKSHINII BAKKA YAALA BAQAQSANII HODHUU HAADHOLII BAQAAQSANII YAALUUN DA’AN WAJJIIN WALQABATAN**, Hospitaaloota wallaggaa bahaatti argaman jedhurratti. Obbo Masfin Abarraa barataa Fayyaa hawaasaa digrii 2ffaa bakka bu’een odeeffannoo dhimma kanaan wal qabatu funaanaan jira.

Kabajamoo hirmaattota keenya, kaayyoon qorannoo kanaa sababoota fi infekshinii bakka yaala baqaqsanii hodhuu wajjiin walqabatan hospitaalota Godina wallaggaa bahatti qorachuu dha. Qorannoo kana keessatti hirmachuuf fedhii kan qabdan yoo ta’e. Gara ibsa gababaa dhimma kanaan walqabatu isiniif kennuutti darbina: erga yaala baqaqsanii hodhuu taasifattanii booda hanga guyyaa 30tti odeeffannoo bakka baqaqsiifatani yaalamtan wajjin wal qabatu nuuf laachuutu isini irraa eegama.Baqaqsanii yaluun booda rakkoon biraa kan hin jirre yoo ta’e, guyyaa 30 keessatti hordoffii si’a 4 taasiftu. Kunis dhuma torban 1ffaa, 2ffaa 3ffaa fi 4ffaa yeroo ta’u, hordoffif kan dhufuu hin dandeenye yoo ta’e bilbila keessani irratti isiniif bilbiluu ni dandeenya. Akkasumas galmee keessan irraa odeeffannoo ni fudhanna. Odeeffannoon isin irraa argamu iccitiin kan qabamu ta’a.

Qorannoo kana keessatti hirmaachuu keessanin miidhaan isin irra gahu tokkollee hin jiru. Odeeffannoo fudhannu keessatti maqaa keessan ibsuun hin barbaachisu.Bu’aan qoranichaas akka waliigalatti malee dhuunfaan hin ibsamu. Qorannoo kanarratti hirmaachuufis ta’e, dhiisuufis mirga guutuu qabdu. Gaaffii ifa isiniif hin taane yoo jiraate gaafachuuf mirga qabdu. Gaaffii deebisuu hin barbaannne deebisuu dhiisuu akkasumas gaaffilee jidduudhaan addaan kutuufis mirga guutuu qabdu.

Qorannoo kana keessatti hirmaachuun faayidaa addaa isiniif hin qabu, garuu odeeffannoo isin kennitan qorannoon Kun galmaa isaa akka qaqqabu ni gargaara akkasumas bu’aan qorannoo kanaas qaama dhimmi isaa ilaallatu garagaraa bira ni dhaqqaba, rakkoo jiru illee ni fura jenne abdanna.Gaaffiwwaan kan xumuruuf daqiiqaa 30 ol hin fudhatu. Kanaafuu, gaaffi armaan gadii kana akka nuuf deebistan kabajaan isin gaafanna. Yeroo keessan qaalii nuuf laattanii,odeeffannoo nuuf laattaniif guddaa galatooma .Yoo gaaffiii qabaattan sodaa tokko malee abbaa qorannichaa lakk. **0917361097/0939344451** bilbilaan gaafachuu ni dandeessu.

Participant contact number-------------------------------------/-------------------------Hospital………………..

Maqaa odeeffannoo funaanaa………………….Mallattoo…………… guyyaa……………

**SSI Data Collection Tool**

**Table 3- Data Collection Tool for assessing incidence and predictors of SSI after Cesarean Deliveries in East Wallaga puplic hospitals, 2024**

| 1. **Socio demographic factors** | | | **Remark** | |
| --- | --- | --- | --- | --- |
| **Code---------------------------** | | |  | |
|  | What is Age of participant? | ---------------Years |  | |
|  | What is Marital status of participant? | 1. Single/Cohabit 2. Married 3. Divorced 4. Widowed |  | |
|  | What is Educational status of participant? | 1. Cannot read & write 2. Can read & write 3. Primary school 4. Secondary school 5. College/university and above |  | |
|  | What is Residence area of participant? | 1. Urban 2. Rural |  | |
|  | What is Ethnicity of participant? | 1. Oromo 2. Amhara 3. Tigrie 4. Guragie 99. Other |  | |
|  | What is Religious of participant? | 1. Orthodox 2. Protestant 3. Muslim 4. Waqefata 99. Other |  | |
|  | What is Occupational status of participant? | 1. Farmer 2. House hold 3. Daily laborer   4. Government employee 5. Merchant 6. NGO worker 7. Student 99. Other |  | |
|  | What is Weight of participant? | ---------------Kg |  | |
|  | What is Height of participant? | ---------------cm |  | |
|  | 1. **Behavioral characteristics** | |  | |
|  | Cigarette or cigar smoker | 1. Nonsmoker 2. Ex-smoker 3. Current smoker |  | |
|  | Does the participant consume alcohol? | 1. Yes 2. No | If no skip to Q12 | |
|  | If yes alcohol consumption Pattern | 1. Occasionally 2. Regularly |  | |
|  | Khat chewing | 1. Yes 2. No |  | |
|  | 1. **Medical related factors** | |  | |
|  | Does participant diagnosed with Diabetes mellitus? | 1. Yes 2. No |  | |
|  | If yes when it diagnosed? | 1. Before pregnancy 2. During pregnancy | If no skip to Q15 | |
|  | Does participant diagnosed with Hypertension? | 1. Yes 2. No |  | |
|  | If yes when it diagnosed? | 1. Before pregnancy 2. During pregnancy | If no skip to Q18 | |
|  | Does participant have any other disease (Comorbidity ) | 1. Yes 2. No |  | |
|  | What is HIV status of participant? | 1. Non-reactive 2. Reactive |  | |
|  | If HIV positive, is client on ART? | 1. Yes 2. No | If no skip to Q21 | |
|  | Is patient on anti-TB treatment? | 1. Yes 2. No |  | |
|  | Thickness of subcutaneous tissue | 1. ≤2cm 2. >2cm |  | |
|  | ASA score: category | 1. I 2. II 3. III 4. IV 5.V |  | |
|  | Post-operative hgb | --------------------------------g/dl |  | |
| 1. **Pregnancy/Intra partum related factors** | | |  | |
|  | Does participant has Antenatal care (ANC) follows up? | 1. Yes 2. No | If No skip to Q28 | |
|  | If yes, ANC booking start at how many weeks? | ----------------------------------------weeks |  | |
|  | Number of ANC contact | 1. 1 time 2. 2–4 times 3. ≥5 times |  | |
|  | Parity | ------------------------------------in number |  | |
|  | Previous cesarean section | 1. Yes 2. No | If No skip to Q31 | |
|  | If yes, how many | ------------------------------------in number |  | |
|  | Gestational age at C/S | ----------------------------------------weeks |  | |
|  | Partograph use | 1. Yes 2. No |  | |
|  | Mother has been in labor before operation | 1. No labor 2. <24hrs 3. >24hrs |  | |
|  | Vaginal Examination done | 1. No exam 2. 1–5 times 3. > 6 times |  | |
|  | postpartum hemorrhage | 1. Yes 2. No |  | |
|  | Membrane state | 1. Intact 2. Ruptured | If Intact skip to Q38 | |
|  | If yes, duration of membrane rupture | 1. <24hrs 2. >24hrs |  | |
|  | Chorioaminonitis | 1. Yes 2. No |  | |
|  | Meconium | 1. Yes 2. No |  | |
|  | Indications | 1. Preeclampsia/eclampsia 2. Uterine rupture 3. Antepartum hemorrhage 4. Previous cesarean section 5. Multiple gestation 6. Cord prolapse 7. Fetal-distress 8. Mal presentation 9. Cephalo pelvic Disproportion (CPD) 10. Grade 3 Meconium Stained Amniotic Fluid 11. The prolonged latent second stage of labour 12. Arrest of Cervical dilatation 13. IUFD 14. Other |  | |
|  | 1. **Procedure related factors** | |  | |
|  | Date of admission | ___ /___/____ |  | |
|  | Date of procedure | ___ /___/____ |  | |
|  | Number of days after admission when procedure was done | --------------in days |  | |
|  | where the procedure performed | 1. Tertiary hospital 2. Primary hospital |  | |
|  | Skin preparation | 1. Iodine 2. Alcohol 3. Chlorohexdine |  | |
|  | Type of surgery | 1. Emergence 2. Elective |  | |
|  | Surgical site Hair removal | 1. Yes 2. No | If no skip to Q49 | |
|  | If yes, where? | 1. Ward 2 . Anesthetic room 3. On the operating table 4. Home |  | |
|  | Type of skin incision | 1. Pfannensteil 2. Midline |  | |
|  | Duration of surgery | ----------------------------------minutes |  | |
|  | The operation is performed by | 1. Resident ≤3 Years 2. Resident >4 Years   3. Gynecologist 4. IESO |  | |
|  | Skin closure | 1. Interrupted sutures 2. Continuous 3.matress |  | |
|  | Skin closed by | 1. Absorable 2. Non absorable |  | |
|  | Surgical wound classification for caesarean section. | 1.Class I: Clean   - Caesarean Section, elective, no pre-rupture of membranes or trial of labour   2. Class II: Clean Contaminated   - Caesarean Section, emergency involving pre-rupture of membranes less than 12hours and /or trial of labour   3. Class III: Contaminated   - Rupture of membranes more than 24hours   4. Class IV: Dirty   - Purulent amniotic fluid |  | |
| 54 | Post-partum hemorrhage | 1. Yes 2. No |  | |
| 1. **Medication factors** | | | | |
| 55 | Antibiotic prophylactic given | 1. Yes 2. No | | If no skip to Q57 |
| 56 | If yes, time of antibiotic prophylactic given before skin incision | ---------------------------------------minutes | |  |
| 57 | Use of antibiotic post-surgery | 1. Yes 2. No | | If no skip to Q59 |
| 58 | If yes, for how long did taken? | -----------------------------in hours | |  |
| 59 | Did patient receive blood transfusions? | 1. Yes 2. No | |  |
| 60 | Type of anesthesia used? | 1. General 2. Spinal 3. Regional/Local 4. Epidural | |  |
| 61 Post Discharge Surveillance | | | | |
|  | State presence of any of the following infection symptoms during inpatient stay | 1. Purulent drainage from the incision Yes No 2. Wound dehiscence Yes 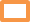 No 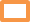 3. Presence of at least one of the following signs or symptoms of infection: 4. Pain or tenderness at operation site Yes 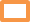 No 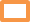 Localized swelling Yes 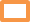 No 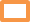 5. Redness Yes 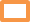 No 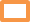 6. Fever (>38°C) Yes 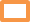 No 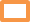 7. Hotness of skin Yes 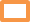 No 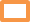   Date of onset of symptoms: ……………………  Date patient discharged in days | | |
|  | Review End of Week 1: Post Discharge Day 4-10  Is patient experiencing any of the following infection symptoms: | 1. Pain/tenderness at operation site Yes 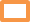 No 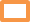 2. Purulent discharge at wound site Yes 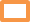 No 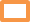 3. Wound dehiscence Yes 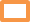 No 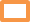 4. Localized swelling Yes 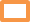 No 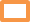 5. Redness Yes 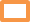 No 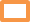 6. Hotness of skin Yes 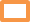 No 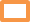   Date of onset of symptoms………………  Outcome ………………………………-----------------------------in days | | |
|  | Review week 2: Post discharge Day 11 – 17 Is patient experiencing any of the following infection symptoms | 1. Pain/tenderness at operation site Yes 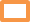 No 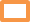 2. Purulent discharge at wound site Yes 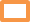 No 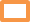 3. Wound dehiscence Yes 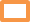 No 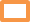 4. Localized swelling Yes 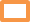 No 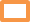 5. Redness Yes 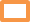 No 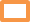 6. Hotness of skin Yes 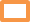 No 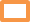   Date of onset of symptoms………………  Outcome ………………………………-----------------------------in days | | |
|  | Review week 3: Post discharge Day 18 – 24 Is patient experiencing any of the following infection symptoms | 1. Pain/tenderness at operation site Yes 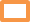 No 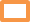 2. Purulent discharge at wound site Yes 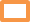 No 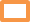 3. Wound dehiscence Yes 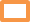 No 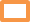 4. Localized swelling Yes 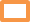 No 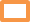 5. Redness Yes 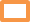 No 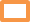 6. Hotness of skin Yes 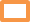 No 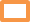   Date of onset of symptoms………………  Outcome ………………………………-----------------------------in days | | |
|  | Review week 4: Post discharge Day 24 - 30  Is patient experiencing any of the following infection symptoms: | 1. Pain/tenderness at operation site Yes 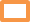 No 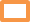 2. Purulent discharge at wound site Yes 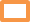 No 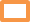 3. Wound dehiscence Yes 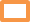 No 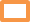 4. Localized swelling Yes 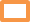 No 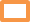 5. Redness Yes 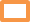 No 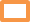 6. Hotness of skin Yes 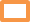 No 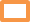   Date of onset of symptoms………………  Outcome ………………………………-----------------------------in days | | |
| 62 | Was a SSI identified within 30 days of procedure? | 1. Yes 2. No | | |
| 63 | If yes, Date SSI detected | ____________________________days | | |
| 64 | If yes ,Type of SSI (Encircle answer) | 1. Superficial 2. Deep 3. Organ/Space | | |
| 65 | What setting | 1. Initial stay 2. Post discharge | | |
| 66 | Follow up completion status | 1. Follow up completed 2. Loss of follow up after some contacts 3. No contact after discharge 4. death | | |

Form completed by ---------------------------- Signature------------------------- Date………………..
