# Supplementary material for: Predictors of surgical site infections following cesarean delivery in public hospitals of West Ethiopia: A cohort study
Source: PLoS One. 2026 Jan 21;21(1):e0339930. doi: 10.1371/journal.pone.0339930 (PMC12822956; doi:10.1371/journal.pone.0339930)
Supplement: S2 File — (DOCX) [file pone.0339930.s002.docx]

Table 1: Sample size determination variables and corresponding sample size of SSI at East Wollega public Hospitals between January 15/2024 to April 14/2024

| **Exposure** | HR | Z_α/2_ | Zβ | Event | Pr(E) | Sample size | Final sample | Reference |
| --- | --- | --- | --- | --- | --- | --- | --- | --- |
| **Multiple vaginal examination** | **1.88** | **1.96** | **0.84** | **44** | **0.25** | **355** | **408** | ([27](#_ENREF_27)) |
| No ANC care | 2.16 | 1.96 | 0.84 | 10 | 0.25 | 246 | 283 | ([27](#_ENREF_27)) |
| Type of incision | 2.6 | 1.96 | 0.84 | 8 | 0.25 | 156 | 179 | ([27](#_ENREF_27)) |
| The operation was carried out by an intern or junior doctor | 4 | 1.96 | 0.84 | 8 | 0.10 | 217 | 250 | ([6](#_ENREF_6)) |

Table 2: Global test of proportional hazards assumption for predictors of SSI among mothers underwent Cesarean Delivery at East Wollega Public Hospitals, between January 15/2024 to April 14/2024

| Predictors | rho | chi2 | df | Prob>chi2 |
| --- | --- | --- | --- | --- |
| Age of participant | -0.09853 | 1.10 | 1 | 0.2938 |
| Educational status | -0.04255 | 0.19 | 1 | 0.6590 |
| Comorbidity | 0.09474 | 1.03 | 1 | 0.3098 |
| ASA score | -0.00263 | 0.00 | 1 | 0.9736 |
| Post-operative hemoglobin | 0.04517 | 0.27 | 1 | 0.6006 |
| Antenatal care (ANC) follows up | -0.10450 | 1.00 | 1 | 0.3164 |
| parity | 0.06925 | 0.65 | 1 | 0.4196 |
| Gestational age | 0.09514 | 1.16 | 1 | 0.2807 |
| Vaginal Examination | -0.00545 | 0.00 | 1 | 0.9511 |
| Chorioaminitis | 0.00172 | 0.00 | 1 | 0.9860 |
| Meconium | 0.09244 | 1.35 | 1 | 0.2447 |
| Type of surgery | -0.05477 | 0.34 | 1 | 0.5613 |
| Surgical wound classification for cesarean section | -0.02038 | 0.05 | 1 | 0.8306 |
| Post-partum hemorrhage | 0.19483 | 1.67 | 1 | 0.0573 |
| Antibiotic prophylactic | -0.03439 | 0.15 | 1 | 0.6978 |
| Blood transfusions | -0.11633 | 1.50 | 1 | 0.2209 |
| Type of anesthesia | -0.00931 | 0.01 | 1 | 0.9202 |
| **Global test** | | **23.19** | **17** | **0.1432** |

Table 3: Life table of Surgical Site Infection among mothers underwent cesarean delivery in East Wollega Public Hospitals

| Interval | Beg. Total | Deaths | Lost | Survival | Std Error | [95% Conf. Int.] | |
| --- | --- | --- | --- | --- | --- | --- | --- |
| 2 3 | 408 | 0 | 2 | 1.0000 | 0.0000 | . | . |
| 3 4 | 406 | 3 | 54 | 0.9921 | 0.0046 | 0.9757 | 0.9974 |
| 4 5 | 349 | 5 | 0 | 0.9779 | 0.0077 | 0.9562 | 0.9889 |
| 5 6 | 344 | 7 | 1 | 0.9579 | 0.0106 | 0.9312 | 0.9744 |
| 6 7 | 336 | 10 | 0 | 0.9294 | 0.0136 | 0.8973 | 0.9518 |
| 7 8 | 326 | 17 | 2 | 0.8808 | 0.0173 | 0.8422 | 0.9105 |
| 8 9 | 307 | 6 | 0 | 0.8636 | 0.0183 | 0.8231 | 0.8954 |
| 9 10 | 301 | 8 | 0 | 0.8406 | 0.0195 | 0.7980 | 0.8750 |
| 10 11 | 293 | 9 | 1 | 0.8148 | 0.0207 | 0.7700 | 0.8517 |
| 11 12 | 283 | 3 | 8 | 0.8060 | 0.0211 | 0.7606 | 0.8437 |
| 12 13 | 272 | 5 | 1 | 0.7912 | 0.0218 | 0.7446 | 0.8302 |
| 13 14 | 266 | 3 | 0 | 0.7823 | 0.0221 | 0.7351 | 0.8220 |
| 14 15 | 263 | 3 | 0 | 0.7733 | 0.0225 | 0.7256 | 0.8138 |
| 15 16 | 260 | 2 | 0 | 0.7674 | 0.0227 | 0.7193 | 0.8084 |
| 18 19 | 258 | 0 | 2 | 0.7674 | 0.0227 | 0.7193 | 0.8084 |
| 24 25 | 256 | 0 | 1 | 0.7674 | 0.0227 | 0.7193 | 0.8084 |
| 30 31 | 255 | 0 | 255 | 0.7674 | 0.0227 | 0.7193 | 0.8084 |
